# Supplementary material for: Beneficial Effect of Mildly Pasteurized Whey Protein on Intestinal Integrity and Innate Defense in Preterm and Near-Term Piglets
Source: Nutrients. 2020 Apr 17;12(4):1125. doi: 10.3390/nu12041125 (PMC7230795; doi:10.3390/nu12041125)
Supplement: Supplementary file 1 [file nutrients-12-01125-s001.zip › Supplementary Figures.pdf]

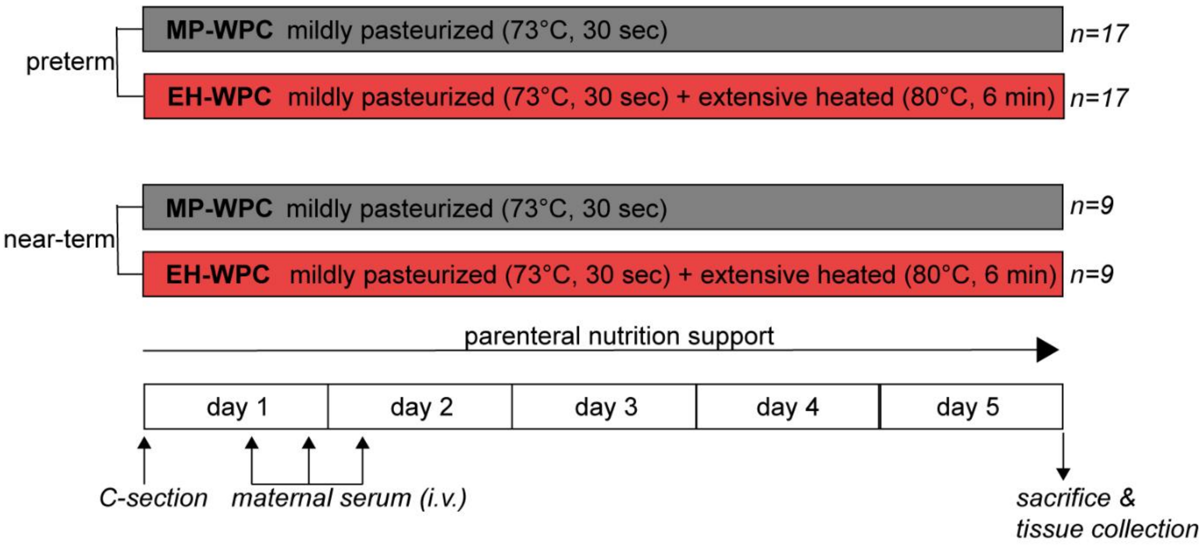

Figure S1: Experimental set-up. Preterm and near-term piglets were delivered by caesarean section at 90% gestation (n=34, 2 litters) and 96% gestation (n=18, 1 litter) respectively. Piglets from each litter were stratified according to birthweight into 2 groups of enteral diets, 1) MP-WPC and 2) EH-WPC. In addition to the enteral nutrition, all piglets received parenteral nutrition support from day 1 to day 5. For passive immunization, piglets received three doses of maternal serum (iv). On day 5 the piglets were euthanized, followed by macroscopic evaluation of gastroenterocolitis and collection of intestinal tissues.

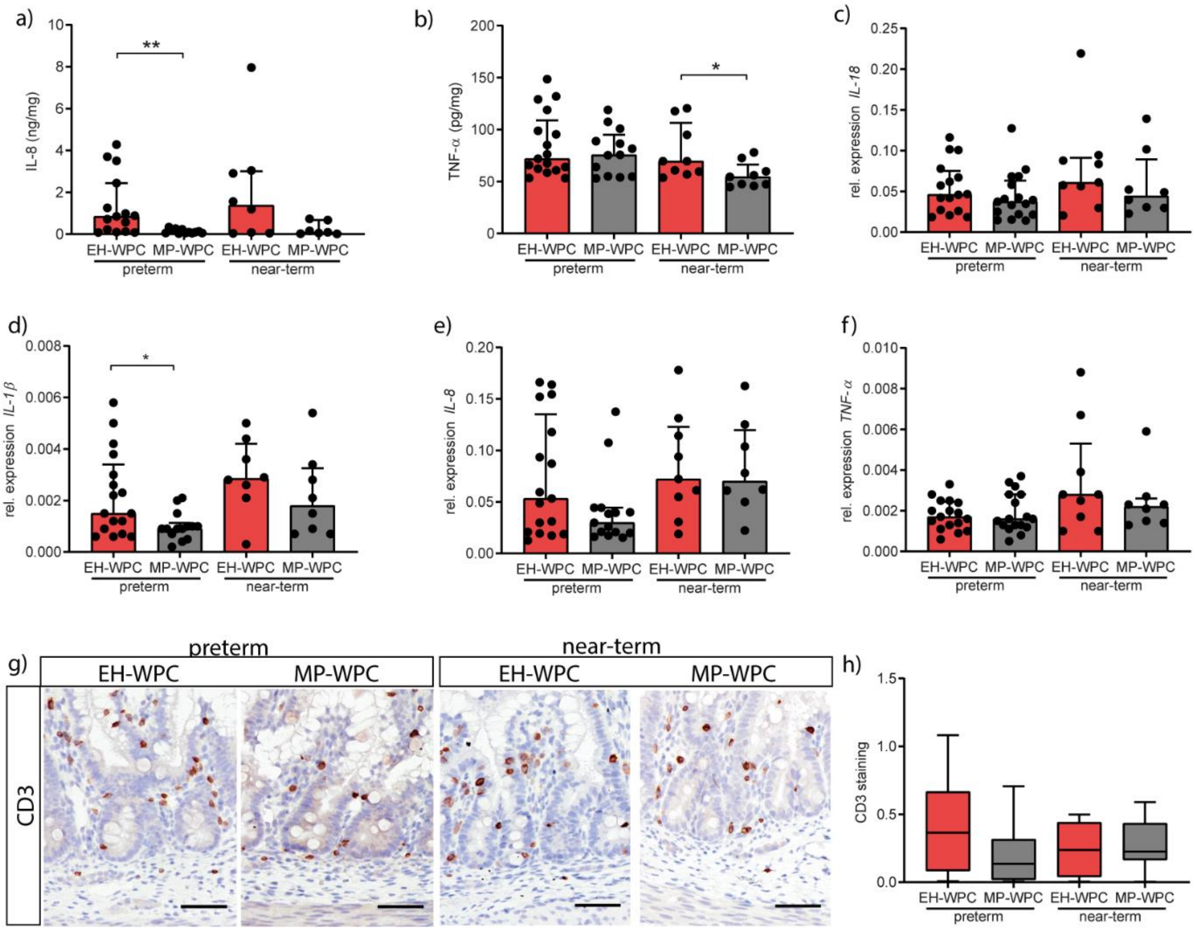

Figure S2: Distal small intestine and colonic inflammation. Cytokine levels of (**a**) IL-8 and (**b**) TNF-α in colon tissue homogenates as determined by ELISA, and relative expression levels of (**c**) *IL-18* in colon tissue as determined by RT-qPCR. Relative expression levels of pro-inflammatory cytokines (**d**) *IL-1β*, (**e**) *IL-8* and (**f**) *TNF-α* in the distal small intestine as determined by RT-qPCR. (**g**) T cells were detected in distal small intestinal tissue by immunohistochemistry staining for CD3, and (**h**) staining intensity in the crypt region was quantified by ImageJ analysis. Values are median ±IQR, *p≤0.05 **p≤0.01. Scalebar equals 50 µm.

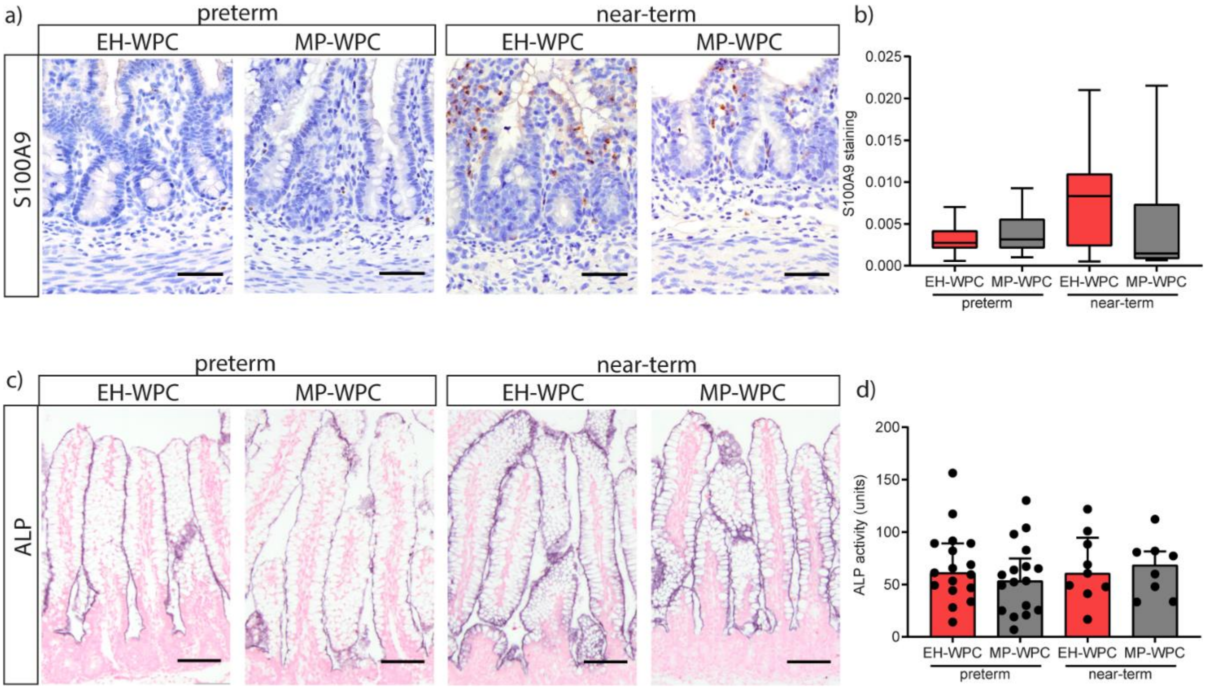

Figure S3: Innate defense in the immature distal small intestine. (**a**) detection of S100A9 by immunohistochemistry in distal small intestinal tissue, with (**b**) relative staining intensity determined by ImageJ analysis. iALP brush border activity was determined with (**c**) NBT/BCIP conversion on distal small intestinal tissue slides and (**d**) pNPP conversion (µg pNPP·mg protein·min^{-1}) in distal small intestinal tissue homogenates. Values are median ±IQR. Scalebar equals 50 µm for (a) and 100 µm for (c).

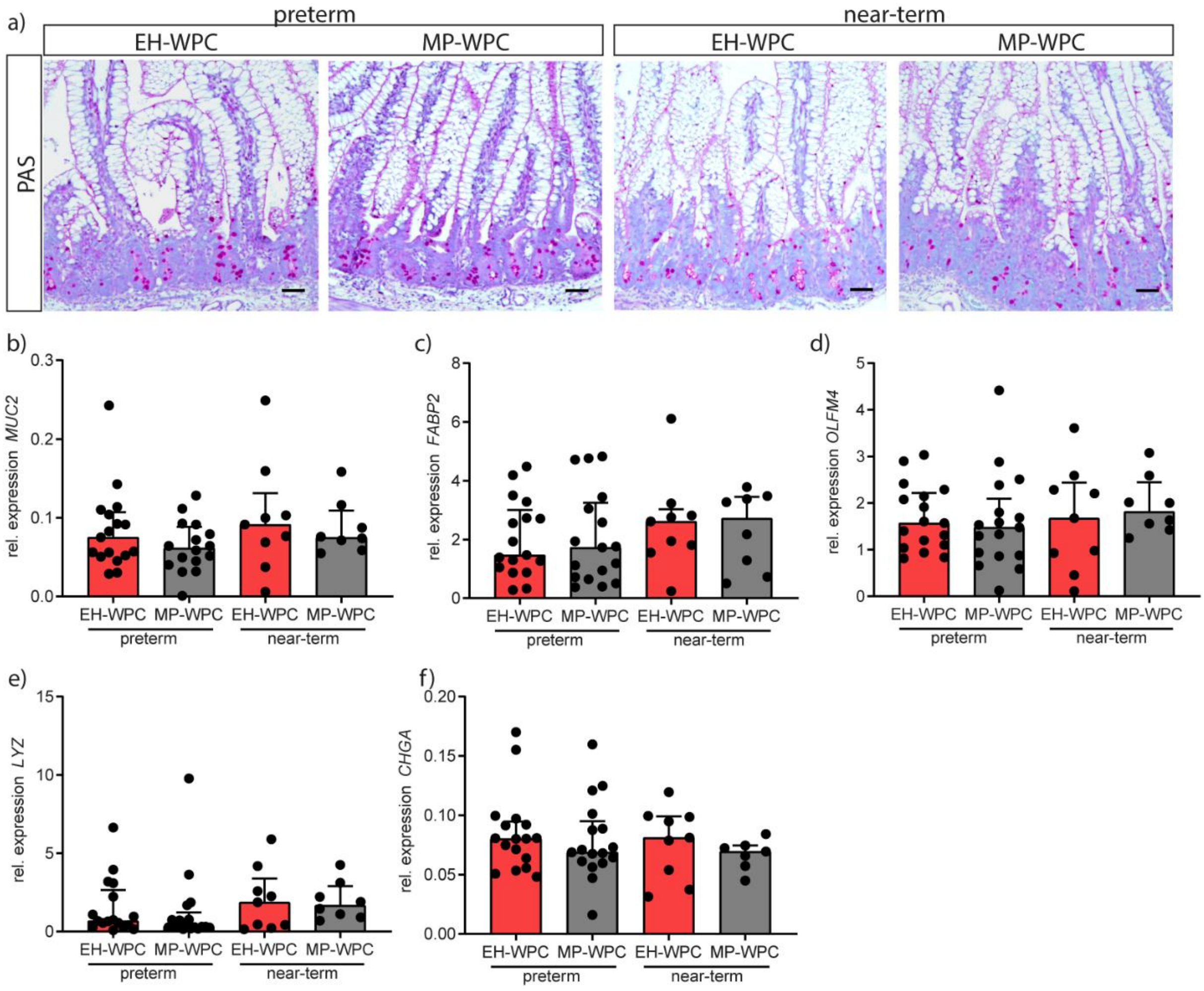

Figure S4: Epithelial cell types in the immature small intestine. (**a**) detection of mucin-containing cells in distal small intestinal tissue by PAS staining, with relative expression levels of (**b**) *MUC2* in distal small intestine, as determined by RT-qPCR. Relative expression levels of enterocyte marker (**c**) *FABP2,* stem cell marker (**d**) *OLFM4*, paneth cell marker (**e**) *LYZ* and enteroendocrine cell marker (**f**) *CHGA* in the distal small intestine as determined by RT-qPCR. Values are median ±IQR, scalebar equals 50 µm.
