# Supplementary material for: Beneficial Effect of Mildly Pasteurized Whey Protein on Intestinal Integrity and Innate Defense in Preterm and Near-Term Piglets
Source: Nutrients. 2020 Apr 17;12(4):1125. doi: 10.3390/nu12041125 (PMC7230795; doi:10.3390/nu12041125)
Supplement: Supplementary file 1 [file nutrients-12-01125-s001.zip › Supplementary Tables.pdf]

Table S1: Macroscopic parameters evaluated to assess severity of gastroenterocolitis.

Score	Macroscopic lesions
1	No or minimal hyperaemic gastroenterocolitis
2	Mild focal gastroenterocolitis
3	Moderate local extensive gastroenterocolitis
4	Severe focal gastroenterocolitis
5	Severe local extensive haemorrhagic and necrotizing gastroenterocolitis
6	Extensive haemorrhagic and necrotizing gastroenterocolitis

Table S2: Parameters evaluated to obtain total histology score. Score of all 5 parameters was added up to obtain a total histology score ranging from 0-15 for the small intestine. For the colon, villus atrophy was not included and score of remaining 4 parameters was added up to obtain a total histology score (0-12).

score	Edema	Epithelial damage	Erythrocytes	Infiltration	Villus atrophy
0 = none	none	Intact epithelium	absent / normal	none	Normal villi
1 = mild	some in lamina propria	Flattened / disorganized	increased in lamina propria	between crypts	Blunted villi
2 = moderate	throughout lamina propria and/or villi	Disrupted / discontinued	Patchy between crypts	in lamina propria	Short & thick villi
3 = severe	extreme	Loss of epithelium	extreme, througouht	transmural	Flat & absent villi

Table S3: List of qPCR primers used

Gene	Forward primer (5'-3')	Reverse primer (5'-3')	NM number
ACTB	CACGCCATCCTGCGTCTGGA	AGCACCGTGTTGGCGTAGAG	XM_021086047
GAPDH	ACACTCACTCTTCTACCTTTG	CAAATTCATTGTCGTACCAG	NM_001206359.1
GUSB	TAACAAGCACGAGGATGCAG	TCCTCTGCGTAGGGGTAGTG	NM_001123121
HPRT1	GGACTTGAATCATGTTTGTG	CAGATGTTTCCAAACTCAAC	NM_001032376.2
RPL4	CAAGAGTAACTACAACCTTC	GAACTCTACGATGAATCTTC	XM_005659862.3
RPL19	GCTTGCCTCCAGTGTCCTC	GCGTTGGCGATTTCATTAG	XM_003131509
CD14	CCTCAGACTCCGTAATGTG	CCGGGATTGTCAGATAGG	NM_001097445.2
CHGA	AAGTCATTGCCCTCCCTGTG	AGATGACCTCGACGATGCAC	NM_001164005.2
FABP2	TGAATCAGCTGGAGACTATGG	TTTACCACGTTAATACCCATTTTT	NM_001031780.1
iALP	TCCCAGACATACAACGTGGA	GGTCTGGTAGTTGGCCTTGA	XM_003133729.4
IL1β	GAAGTGATGGCTAACTACGGTGAC	TCTCAGAGAACCAAGGTCCAGGT	NM_214055.1
IL6	TGGCTACTGCCTTCCCTACC	CAGAGATTTTGCCGAGGATG	NM_214399.1
IL8	GAAGAGAACTGAGAAGCAACAACA	TTGTGTTGGCATCTTTACTGAGA	NM_213867.1
IL10	CGTAATGCCGAAGGCAGAGA	CTTGGAGCTTGCTAAAGGCAC	NM_214041.1
IL18	CGATGAAGACCTGGAATCGG	CATCATGTCCAGGAACACTTCTCTG	NM_213997.1
IL22	AGCAGCCCTACATCACCAAC	GCATTAAGAGTAATCAGCAGTACCT	XM_021091967.1
LYZ	GGTCTATGATCGGTGCGAGT	AACTGCTTTGGGTGTCTTGC	NM_214392.2
MUC1	GTGCCGACGAAAGAACTG	TGCCAGGTTCGAGTAAGAG	XM_021089728.1
MUC2	GGCTGCTCATTGAGAGGAGT	ATGTTCCCGAACTCCAAGG	XM_021082584
OLFM4	GTCAGCAAACCGGCTATTGT	TGCCTTGGCCATAGGAAATA	XM_003482903
TNFα	CACGTTGTAGCCAATGTCAAAG	GAGGTACAGCCCATCTGTCG	NM_214022.1
TLR4	CAGATAAGCGAGGCCGTCATT	TTGCAGCCCACAAAAAGCA	NM_001293316
VIL1	TGGGGACAAAGGTCGCT	TGCCATAGGTGCTGGAAGAAA	XM_001925167.6

Table S4: Clinical characteristics of preterm and near-term piglets on EH-WPC or MP-WPC diet for 5 days. Values are median ±IQR, medians not sharing the same superscript letter differ significantly ($p \leq 0.05$).

	Preterm piglets		Near-term piglets	
	EH-WPC	MP-WPC	EH-WPC	MP-WPC
n	17	17	9	8
Birthweight (g)	955 ± 369 ^{a}	942 ± 287 ^{a}	1183 ± 495 ^{b}	1184 ± 475 ^{b}
Drop-outs (n)	3	3	-	-
Birthweight (g) *excl. drop-outs*	941 ± 405 ^{a}	919 ± 272 ^{a}	-	-
Weight day 5 (g) *excl. drop-outs*	1067 ± 484 ^{a}	1015 ± 394 ^{a}	1356 ± 638 ^{b}	1328 ± 558 ^{b}
Increase bodyweight (g/kg·day^{-1}) *excl. drop-outs*	2.1 ± 2.1 ^{a}	2.0 ± 1.6 ^{a}	3.6 ± 1.5 ^{b}	2.9 ± 0.8 ^{b}
Increase bodyweight (%) *excl. drop-outs*	11 ± 9.8 ^{a}	10 ± 7.3 ^{a}	18 ± 7.5 ^{b}	15 ± 3.8 ^{b}
Standing (hrs after birth) *excl. drop-outs*	25 ± 8 ^{a}	26 ± 9 ^{a}	11 ± 16 ^{b}	5 ± 13 ^{b}
Liver (g/kg) *excl. drop-outs*	29 ± 4.7 ^{a}	30 ± 5.3 ^{a}	30 ± 5.5 ^{a}	29 ± 4.7 ^{a}
Spleen (g/kg) *excl. drop-outs*	2.1 ± 1.0 ^{a}	1.5 ± 0.7 ^{b}	2.3 ± 0.7 ^{a}	2.1 ± 0.8 ^{a}
Stomach (g/kg) *excl. drop-outs*	7.3 ± 2.9 ^{a}	6.4 ± 2.1 ^{a}	7.3 ± 3.2 ^{a}	5.5 ± 1.1 ^{b}
Small intestine (g/kg) *excl. drop-outs*	31 ± 4.3 ^{a}	32 ± 6.1 ^{a}	30 ± 11 ^{a}	29 ± 3.0 ^{a}
Colon (g/kg) *excl. drop-outs*	12 ± 2.0 ^{a}	11 ± 3.1 ^{a}	12 ± 2.1 ^{a}	11 ± 1.5 ^{a}
